# Impact of anterior callosal disconnection on picture naming in frontal lobe epilepsy surgery

**DOI:** 10.1093/braincomms/fcaf317

**Published:** 2025-09-03

**Authors:** Davide Giampiccolo, Lawrence P Binding, Umesh Vivekananda, Zara Fenlon, Roman Rodionov, Jane de Tisi, Fenglai Xiao, Aidan O’Keeffe, Andrew W McEvoy, Fahmida A Chowdhury, Sallie A Baxendale, Anna Miserocchi, John S Duncan

**Affiliations:** Department of Clinical and Experimental Epilepsy, UCL Queen Square Institute of Neurology, University College London, London, WC1N 3BG, UK; Victor Horsley Department of Neurosurgery, National Hospital for Neurology and Neurosurgery, London, WC1N 3BG, UK; Institute of Neuroscience, Cleveland Clinic London, London, 33 Grosvenor Pl, SW1X 7HY, UK; Department of Clinical and Experimental Epilepsy, UCL Queen Square Institute of Neurology, University College London, London, WC1N 3BG, UK; Department of Computer Science, Centre for Medical Image Computing, UCL, London, WC1V 6LJ, UK; Department of Clinical and Experimental Epilepsy, UCL Queen Square Institute of Neurology, University College London, London, WC1N 3BG, UK; Department of Clinical and Experimental Epilepsy, UCL Queen Square Institute of Neurology, University College London, London, WC1N 3BG, UK; Department of Clinical and Experimental Epilepsy, UCL Queen Square Institute of Neurology, University College London, London, WC1N 3BG, UK; Department of Clinical and Experimental Epilepsy, UCL Queen Square Institute of Neurology, University College London, London, WC1N 3BG, UK; Department of Clinical and Experimental Epilepsy, UCL Queen Square Institute of Neurology, University College London, London, WC1N 3BG, UK; School of Mathematical Sciences, University of Nottingham, Nottingham, NG7 2RD, UK; Institute of Epidemiology and Health Care, UCL, London, WC1E 7HB, UK; Department of Clinical and Experimental Epilepsy, UCL Queen Square Institute of Neurology, University College London, London, WC1N 3BG, UK; Victor Horsley Department of Neurosurgery, National Hospital for Neurology and Neurosurgery, London, WC1N 3BG, UK; Institute of Neuroscience, Cleveland Clinic London, London, 33 Grosvenor Pl, SW1X 7HY, UK; Department of Clinical and Experimental Epilepsy, UCL Queen Square Institute of Neurology, University College London, London, WC1N 3BG, UK; Department of Clinical and Experimental Epilepsy, UCL Queen Square Institute of Neurology, University College London, London, WC1N 3BG, UK; Department of Clinical and Experimental Epilepsy, UCL Queen Square Institute of Neurology, University College London, London, WC1N 3BG, UK; Victor Horsley Department of Neurosurgery, National Hospital for Neurology and Neurosurgery, London, WC1N 3BG, UK; Institute of Neuroscience, Cleveland Clinic London, London, 33 Grosvenor Pl, SW1X 7HY, UK; Department of Clinical and Experimental Epilepsy, UCL Queen Square Institute of Neurology, University College London, London, WC1N 3BG, UK

**Keywords:** epilepsy, disconnection, language, epilepsy surgery, frontal lobe

## Abstract

Epilepsy surgery in focal, drug-resistant frontal lobe epilepsy can be curative and resection is aimed at seizure freedom. The cognitive impact of surgery, however, is less clear-cut. On one hand, resection of the epileptogenic zone can disconnect essential brain networks and therefore cause dysfunction. On the other hand, surgery may prompt recovery of normal brain function by restoring normal electrical activity as propagating epileptic discharges affect cognition outside the epileptogenic zone. To understand the impact of surgery on cognitive outcome, we investigated picture naming in 51 patients undergoing frontal lobe epilepsy surgery (28 left-hemisphere-dominant; 23 language-dominant) preoperatively and at 12 months follow-up using complementary voxel-based lesion symptom mapping, tractwise voxel-based disconnectome and tractography analyses to investigate cortical regions and white matter structures associated with language performance. Naming performance significantly improved 1 year after surgery compared with preoperatively, irrespective of the operated hemisphere or dominance. Improved naming performance was associated with freedom from seizures with impaired awareness. No damage to any region or white matter structure was associated with language decline. Voxel-based disconnectome analysis identified a region in the anterior corpus callosum associated with improved naming. This was confirmed by the tractography disconnectome analysis showing that naming improvement was linked to anterior callosal disconnection between regions linking presupplementary/supplementary motor areas, posterior middle frontal and inferior frontal gyri bilaterally. Our results suggest that seizure reduction can underlie language improvement: in line with results from hemispherotomy or callosotomy, isolating epileptic activity from the language network through callosal disconnection may support cognitive recovery.

## Teaser

Giampiccolo *et al.* showed that in individuals having neurosurgery for drug-resistant frontal lobe epilepsy, naming performance can improve if epileptogenic networks including the corpus callosum are disconnected.

## Graphical Abstract

**Graphical Abstract:**
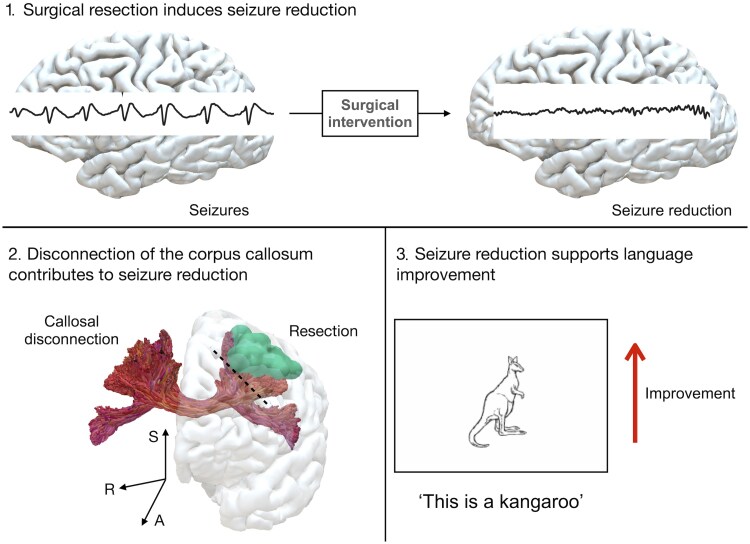

## Introduction

In drug-resistant focal frontal lobe epilepsy seizure freedom is achieved in more than half of operated patients, with an additional 25% experiencing decreased seizures.^1,2^ The impact surgery has on cognition, however, is less clear-cut. While resection of the epileptic focus subtends seizure freedom, it also interrupts propagation of ictal and inter-ictal discharges.^3^ Abnormal electrical discharges impair function,^4^ and neuropsychological impairments are common in epilepsy patients in the absence of structural lesions.^5^ This may suggest two different scenarios. On one hand, cortico-subcortical structures involved in cognition may be part of the epileptogenic zone and therefore their disconnection would cause a behavioural deficit. Conversely, in those individuals in whom function lies outside the epileptogenic zone and is impacted by propagated epileptic discharges, excision of the epileptogenic zone—or disconnection of the epileptogenic network—may restore function by impeding propagation of these disharges.^6^ Studies of cognitive outcome after epilepsy surgery have shown improvement in memory function in 21% of patients after temporal lobe resection^5^ and in language function in 14% of patients after frontal lobe resections.^7^ Importantly, this is the case not only for resective surgery, but also for disconnection. Hemispherotomy can enhance cognition while abolishing seizures, with language improving, especially in patients with a preoperative deficit.^8^ Similarly, callosotomy can improve motor control irrespective of postoperative seizure outcome.^9^ This suggests that terminating seizures or isolating the epileptogenic network from the language network may improve language outcome.

Nevertheless, resection can also cause dysfunction. The current understanding of language has questioned classical language cortices such as Broca’s or Wernicke’s area and involves two streams:^10,11^ a dorsal stream focused on production of speech (arcuate fasciculus/third branch of the superior longitudinal fasciculus),^12^ and a ventral stream focused on language content (inferior fronto-occipital fasciculus/inferior longitudinal fasciculus).^13-15^ There is growing evidence from stroke and neurosurgery literature that language deficits arise from subcortical disconnection of these essential white matter pathways, and conditions such as non-fluent aphasia,^12^ conduction aphasia,^12^ semantic aphasia,^13,16^ anomia,^14^ or jargon aphasia^17^ may represent disconnection syndromes. Thus, disconnection of the epileptogenic network may enable improvement of language function, provided essential language connections are spared.

At present, the impact of frontal lobe resection on language outcome is unclear. Transecting the epileptogenic network may improve language function, but disconnection of critical language pathways may lead to aphasia. To investigate this, we retrospectively reviewed 51 patients with pre- and postoperative language assessments, voxel-lesion symptom mapping, tractwise voxel-based and tractography disconnectome analyses, to evaluate the impact of frontal lobe epilepsy surgery on language and to determine the white matter pathways linked to language outcome.

## Materials and methods

### Participants

The study design was a retrospective case collection. We initially reviewed the clinical data of 125 consecutive patients who underwent surgery for frontal lobe epilepsy at the National Hospital for Neurology and Neurosurgery between 1990 and 2021. Of these, we selected individuals who met the following criteria: (i) available preoperative and at least 3-month postoperative MRI, (ii) preoperative language outcome and at 12-month follow-up. A total of 51 patients were subsequently selected. We evaluated age, sex, epilepsy onset, epilepsy duration, years of education, handedness, lesion side, histology, seizure outcome and resection volume. Seizure outcome was evaluated categorically following the grading of International League against Epilepsy (ILAE classification—1: no seizure; 2: auras, no other seizures; 3: 1–3 seizure days/year, ±auras; 4: four seizure days/year—50% reduction in baseline no. of seizure days; ±auras; 5: <50% reduction in baseline no. of seizure days—100% increase in baseline no. of seizure days; ±auras; 6: >100% increase in baseline no. of seizure days; ±auras),^18^ and binarily into (i) seizure-free (ILAE Grade I; ILAE 1) and free from seizures with impaired awareness (ILAE 2) or (ii) continuing seizures (ILAE 3–6). Considering that patients were operated for ongoing seizures resistant to drugs, ILAE Grades 1 and 2 after surgery were considered as a marked improvement in seizure control. Antiseizure medication (ASM) potentially affecting language such as topiramate and zonisamide were recorded.^19^ The study was conducted in accordance with the ethical standards of the Declaration of Helsinki and approved by the Health Research Authority (Ref: IRB: 22/SC/0016).

### Imaging acquisition and processing

Structural MRI datasets were collected preoperatively and at a minimum follow-up of 3 months on a 1.5-T or a 3-T MR imaging scanner (General Electric, Waukesha, Milwaukee, WI). Scans were acquired with different protocols over time: until 2003 (1.5 Tesla GE Signa Echo-speed scanner): T1-weighted 3D volumes were acquired on a coronal plane using an inversion-recovery prepared fast spoiled gradient recall sequence (GE), TE/TR/NEX 4.2 ms (fat and water in phase)/15.5 ms/1, time of inversion (TI) 450 ms, flip angle 20°, to obtain 124 slices of 1.5 mm thickness with a field of view (FOV) of 18–24 cm with a 192 × 256 matrix, 0.9375 × 0.9375 × 1.5 mm voxel size. We used N3 for non-uniformity correction, and then images were resliced to isotropic voxels of 0.9375 × 0.9375 × 0.9375 mm^3^, while preserving the native resolution. Between 2003–13 (3T GE Signa Excite HDx) and 2014–23 (3T GE Discovery MR750): T1-weighted imaging was 1 mm isovolumetric, performed with inversion-recovery fast spoiled gradient recalled echo [echo time (TE) 3.1 ms, repetition time (TR) = 7.4 ms, inversion time = 400 ms, FOV = 224 × 256 × 256 mm, matrix = 224 × 256 × 256, parallel imaging acceleration factor = 2]. A coronal T2-weighted sequence (TE = 30/119 ms, TR = 7600 ms, FOV = 220 × 220 mm, matrix = 512 × 512, slice thickness = 4 mm, voxel size = 0.43 × 0.43 × 4.00 mm = 0.74 mm^3^, SENSE factor = 2) was also acquired. Postoperative T1 MRI performed at least 3 months after surgery was used to exclude immediate postoperative brain-shift and swelling. Part of the dataset has been published in a previous study.^20^ Diffusion MRI comprised (i) a single-shell acquisition using a cardiac-triggered single-shot spin-echo planar imaging sequence (1.875 × 1.875 × 2.4 mm resolution, 52 directions, 6 b0, *b*-value:1200 s/mm^2^, nine patients) and (ii) a multi-shell acquisition (1.6 mm isotropic resolution, 101 directions, 14 b0, *b*-values: 300, 700 and 2500 s/mm^2^, 12 patients).

### Diffusion MRI preprocessing and processing

Diffusion MRI data were corrected for noise, Gibbs ringing and signal drift using MRTrix3 (https://www.mrtrix.org).^21^ Distortion correction was performed using a synthesized b0 (Synb0-DisCo) produced from a T1-weighted MRI.^22^ The result was then included into FSL’s *Topup*. Magnetic susceptibility field, Eddy current and motion artefact correction were performed using FSL (https://fsl.fmrib.ox.ac.uk/fsl).^23^ Response functions for cerebrospinal fluid, and white and grey matter were estimated using Single-Shell 3-Tissue CSD and Multi-Shell 3-Tissue CSD in MRTrix 3. Anatomically constrained tractography (ACT) using hybrid surface and volume segmentation in MRTrix3 was performed using second-order integration over fibre orientation distribution probabilistic fibre tracking algorithm selecting a maximum of 5000 streamlines from 30 million seeds.^16^

### Neuropsychological assessment

Language lateralization was characterized using language fMRI (verb generation task) in 40 patients. For those patients in whom language fMRI was not diagnostic (2 patients) or was not performed (11), language lateralization was evaluated using the Edinburgh Handedness Inventory.^24^ Patients with bilateral language distribution were considered to have dominant language function in both hemispheres as language was shown in both. Picture naming (Graded Naming Test, consisting of oral naming of 30 black and white pictures)^25^ was assessed preoperatively and 12 months after surgery. Naming performance was evaluated on a continuous scale as a *z-*score both preoperatively and postoperatively. *z-*scores were based on recent normative data on the Graded Naming Test.^26^ Considering that patients had multiple assessments before surgery and after surgery and that the Graded Naming Test has shown little practice effect,^27^ no normative correction for practice effects was applied. Further, we also used reliable change indices (RCIs) using a 90% confidence interval^7,28,29^ to evaluate categorically individual variations of language performance and to take into account test-rest effects based on individual performance. Accordingly, if the number of objects named increased by four (90% CI) or more, this was considered to be a significant improvement, if performance worsened by four (90% CI) or more items this was considered to be a decline, as previously.^16,27^

### Language lateralization

Verbal generation fMRI was used to determine patients’ expressive language lateralization for the inclusion criteria in this study, as previously.^16^ Each patient performed a verbal fluency fMRI language task. During the verb generation task, subjects generated verbs associated with a visually-displayed noun (‘Generate’) or repeated a visually-displayed noun (‘Repeat’). There were four 30 s blocks per condition and four cross-hair fixation blocks. Hemispheric language lateralization was calculated using the bootstrap method of the lateralization index toolbox implemented in SPM8 on spmT maps. The WFU PickAtlas’ anatomical masks of the middle and inferior frontal gyri (including the pars triangularis, orbitalis, opercularis) were used based on lateralising reliability of these regions. LI values were calculated: [LI = (L–R)/(L + R)]. Groups were defined by an LI>+0.2 (left-hemisphere-dominant), −0.2 < LI < 0.2 (bilateral) and LI < −0.2 (right-hemisphere-dominant). For verbal generation, fMR gradient-echo planar T2*-weighted images were acquired with 50 contiguous 2.4 mm (0.1 mm gap) slices with a 24 cm FOV, 64 × 64 matrix with an in-plane voxel size of 3.75 × 3.75 mm (TE/TR = 22/2500 ms).

### Resection cavities construction and normalization

Each individual resection cavity was manually drawn from the postoperative MRI using ITKsnap (http://www.itksnap.org). The individual brain anatomy with the related resection cavity was normalized to a template of 152 patients (MNI; Montreal Neurological Institute) using an enantiomorphic normalization from SPM12 (https://www.fil.ion.ucl.ac.uk/spm/software/spm12/).^30^ A flowchart of the image processing can be found in the Supplementary material.

### Voxel-lesion symptom mapping

To determine which resection locations were associated with naming performance we used permutation tests on the normalized resection cavities in FSL’s *Randomize* with 10 000 permutations, family wise error (FWE)-correction and threshold-free cluster enhancement^31^ using *z-*scores variations similar to previous publications.^20,32,33^ Detailed information on this can be found in the Supplementary material.

### Tractwise voxel-based disconnectome analysis

We computed the disconnectome map of each patient using the BCBtoolkit (http://www.bcblab.com). These maps show, for each voxel, the probability of having shared white matter fibres disconnected based on a healthy population tractography dataset.^34,35^ Briefly, tractogram of fibres passing through the resection cavity of healthy right-handed adults from the 7T dataset of the Human Connectome Project were processed using High Angular Resolution Diffusion Imaging tractography (spherical deconvolution). For each patient lesion, 20 tractograms are generated and overlayed to produce a probabilistic map. A sample of 20 was chosen as this accounts for more than 80% of shared variance in the overall population,^34^ and selected a probability of disconnection above the level of chance, as previously.^20,35^ A voxel-based permutation test was performed on disconnection maps using FSL’s *Randomize* with 10 000 permutations, FWE-correction and threshold-free cluster enhancement^31^ on *z-*score variations (postoperative *z-*score subtracted to the preoperative in each subject) to determine which disconnection profiles were associated with language outcome.^20,32,33^

### Tractography analysis

The tractwise voxel-based analysis can highlight locations associated with language performance but cannot indicate the underlying fibre bundles. We therefore performed an additional tractography analysis in 21 patients who had preoperative tractography. White matter bundle reconstruction was performed using probabilistic constrained spherical deconvolution in MRTrix3^16^ focusing on tracts overlapping the results of the tractwise voxel-lesion symptom mapping analysis. This highlighted callosal fibres. Therefore, the corpus callosum was selected: this was dissected and divided in segments (rostrum, genu, rostral body, anterior midbody) following the subdivision proposed by Witelson.^36,37^ A detailed description of the tractography procedure and the regions of interest selected can be found in the Supplementary material.

### Statistical analysis

Statistical analysis was performed using SPSS Statistics (28, IBM, Armonk, NY, USA). For each variable of interest, normality was assessed using a using a Kolmogorov–Smirnov test.

A paired *t*-test was used to assess changes in naming performance using *z-*scores by comparing preoperative naming performance with postoperative performance within a subject with a Holm–Bonferroni method. A multivariable linear regression model was used to analyse changes in postoperative from preoperative naming *z-*scores with age, sex, years of education, resection volume, epilepsy duration and use of topiramate or zonisamide as covariates. A linear regression model was used to evaluate the relationship between postoperative *z-*score and seizure outcome in patients in whom there were no seizures with impaired awareness (ILAE 1 and 2) compared to the others (ILAE 3–6): this analysis was performed using postoperative *z-*scores rather than change in *z-*score from preoperatively, to avoid evaluation bias in patients who were not impaired preoperatively and therefore would not improve.^4,38^ A voxel-based permutation test was performed on disconnection maps using FSL’s *Randomize* with 10 000 permutations, FWE-correction and threshold-free cluster enhancement^31^ on *z-*score variations (postoperative *z-*score subtracted to the preoperative in each subject) to determine which disconnection profiles were associated with language outcome (either language decline or language improvement).^20,32,33^ A linear regression model corrected with Holm–Bonferroni method was used to investigate the association between tract disconnection (postoperative number of streamlines for each component of the corpus callosum minus preoperative number of streamlines for each component of the corpus callosum and then converted into a percentage) and naming outcome (variation in postoperative from preoperative naming *z-*scores), for each callosal component.

## Results

### Participants demographics

**Figure 1:**
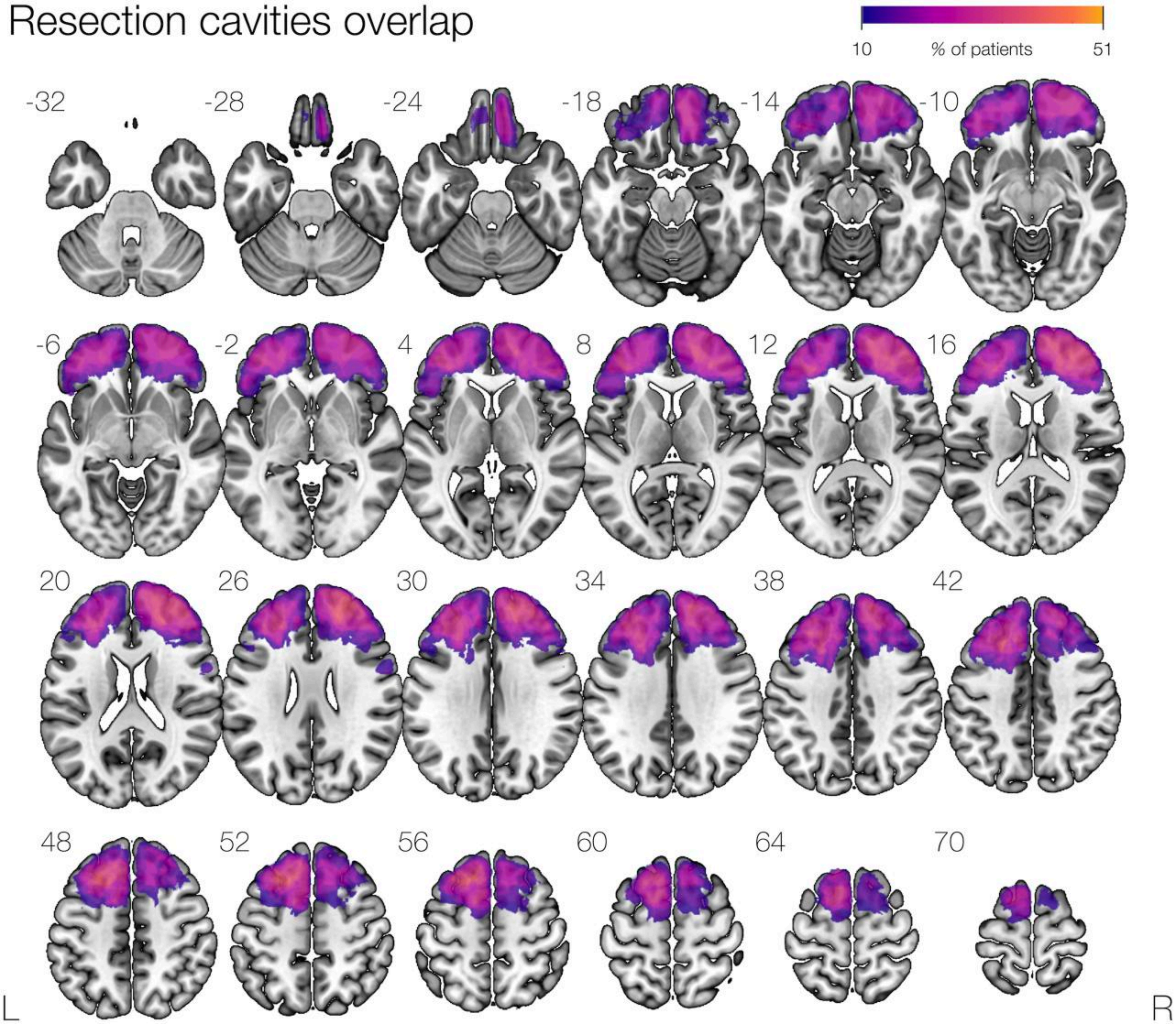
**Overlap map of frontal lobe resection cavities.** A map of all resection cavities normalized to the MNI space is shown, with a colour bar showing voxel overlapping in the cohort of 51 patients.

The demographics of 51 individuals who underwent frontal lobe epilepsy (age: 36 ± 11 years; 24 females, 28 operated in the left hemisphere, 33 lesional) are shown in Table 1. Epilepsy onset was 11 ± 7 years, while epilepsy duration was 25 ± 12 years. An overlap of all resection cavities of the 51 patients is shown in Fig. 1. The most common histology was focal cortical dysplasia (25 patients) followed by dysembryoplastic neuroepithelial tumour (DNT, 5), cavernoma (2), and glioma (Grade I or II) (3). Histopathology showed gliosis or no appreciable disease in the 15 other cases. Of 51, 24 (47%) were seizure-free at 1 year after resection. The median resection volume was 26 cc (interquartile range: 53–12 cc). Six patients were treated with topiramate and eight patients with zonisamide. Only one patient modified ASM during the study period, stopping topiramate before the 1-year assessment.

**Table 1:** Patient demographics

Patient	Age	Sex	Operated hemisphere	Handedness	fMRI dominance	Histology	Medications affecting language	ILAE grade 1 year after surgery
1	28	M	Right	R	L	NAD	no	3
21	39	F	Left	R	L	DNT	no	1
22	22	M	Right	R	both	FCD IIb	no	1
23	35	M	right	R	L	Gliosis	ZNS	1
24	29	M	Left	R	L	FCD IIb	no	1
25	28	F	Right	R	both	FCD IIb	no	1
26	53	F	Left	R	L	DNT	no	5
27	28	F	Right	R	L	CAV	no	1
28	36	F	Left	R	L	NAD	no	5
29	28	F	Left	R	L	FCD IIb	TPR	2
30	39	M	Right	R	L	Gliosis	TPR	5
31	28	M	Left	R	L	Glioma (WHO I)	no	1
32	27	M	Right	R	both	FCD IIa	no	4
33	32	F	Right	R	L	NAD	no	4
34	32	M	Left	R	L	FCD IIb	ZNS	1
35	32	M	Right	R	L	Gliosis	ZNS	3
36	17	M	Left	R	both	NAD	no	2
37	27	F	Left	L	L	FCD IIa	no	1
38	60	F	Left	L	both	FCD IIb	TPR	2
39	22	F	Left	R	L	Gliosis	no	2
40	20	F	Right	R	both	FCD IIb	TPR (discontinued)	1
41	43	M	Right	R	L	Cortical scar	no	3
42	56	M	Left	L	L	FCD IIb	no	1
43	37	M	Left	R	non-diagnostic	FCD	no	5
44	61	F	Left	L	R	FCD	no	8
45	42	F	Left	L	both	FCD	ZNS	1
46	21	M	Right	Both	Not diagnostic	FCD IIb	no	3
47	47	M	Right	R	L	OTHER	no	1
48	30	M	Left	L	L	NAD	ZNS	4
49	34	M	Right	R	L	FCD IIb	no	3
50	42	F	Left	R	L	CAV	no	1
51	47	F	Left	R	both	FCD	ZNS	4

DNT, dysembryoplastic neuroepithelial tumour; FCD, focal cortical dysplasia; NAD, no appreciable disease; TPR, topiramate; ZNS, zonisamidde.

### Language assessment

In total, 40 patients underwent preoperative language fMRI: 26 patients were left language dominant, one was right language dominant and 11 showed bilateral language distribution. About 11 patients did not undergo language fMRI and in two patients, the results were considered non-diagnostic: 12 were right-handed and one ambidextrous. Neuropsychological data are summarized in Table 2.

**Table 2:** Patients’ neuropsychological evaluation

Years of education = 12 ± 1.7 years (mean, SD)	
**Graded naming task**	
**Preoperative (*n*** **=** **51)**	
Number of items	*z-*score
17 ± 4.9	−1.14 ± 1.46
**1-year postoperative (*n*** **=** **51)**	
Number of items	*z-*score
18 ± 4.8	−0.9 ± 1.49
**Preoperative/postoperative performance modifications (*n*** **=** **51)**	
Number of items	*z-*score
1 ± 2.6	+0.4 ± 0.68
**Preoperative/postoperative performance modifications in patients with preoperative impairment (*n*** **=** **18)**	
Number of items	*z-*score
2.3 ± 2.6	+0.7 ± 0.7

Averaged number of correct item per category is shown.

**Figure 2:**
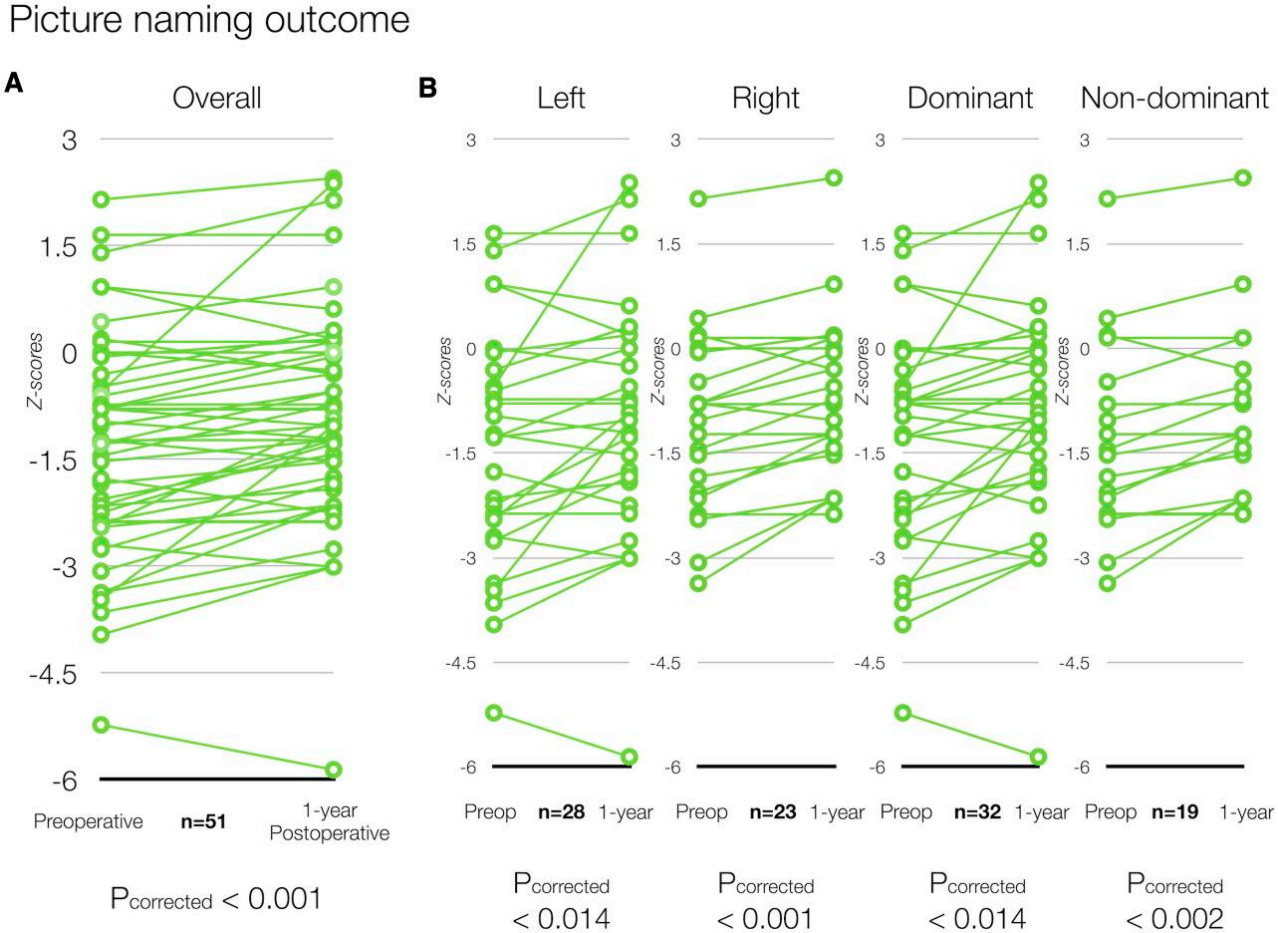
***z*-Score distributions of picture naming performance.** (**A**) Naming performance in the overall cohort is shown contrasting preoperative and 1-year postoperative z-scores using a paired *t*-test. (**B**) Naming performance pre- and postoperatively is shown according to side (left or right) and language dominance (dominant versus non-dominant). Results were adjusted to account for multiple comparisons using the Holm–Bonferroni method.

Considering the full cohort of 51 patients, 33/51 patients (65%, 16L, 17R; raw scores: 17 ± 4.9 items; *z-*scores: −1.14 ± 1.46) had normal naming performance and 18/51 (35%, 12L, 6R) tested were impaired (−1.96 or inferior on *z-*score) preoperatively. At the 12-month follow-up (raw scores: 18 ± 4.8 items; *z-*scores: −0.9 ± 1.49), 40/51 (78.4%, 21L, 19R) performed normally or above normal in picture naming, while 11/51 (21.5%, 7L, 4R) were impaired. Only 1/33 (3%, 1L) patient who had normal language performance showed picture naming decline at 12 months and 8/18 (42%; 6L, 2R) patients with significant preoperative naming deficits improved to a normal range of 12 months after surgery. No patient showed a significant decline in performance as assessed with RCI while 5/51 (4L, 1R) patients had improved. 4/5 (3L, 1R) of patients who improved in RCI were impaired preoperatively when considering the *z-*score. A chart of picture naming *z-*score changes between preoperative and 12-month postoperative scores is shown in Fig. 2.

Picture naming performance significantly improved 12 months after resection (raw score variation: 1 ± 2.6; *z-*score variation: + 0.4 ± 0.68; *t(*50) = −4.202, *p*_corrected_ < 0.001). This occurred irrespective of the operated side [left hemisphere = raw score variation: 1.57 ± 3.32; *z-*score variation: + 0.4 ± 0.8; *t(*27) = −2.624, *p*_corrected_ < 0.014; right hemisphere = raw score variation: 1.3 ± 1.46; *z-*score variation: + 0.37 ± 0.4; *t(*22) = −4.40, *p*_corrected_ < 0.001)] or language dominance (dominant = raw score variation: 1.53 ± 3.14; *z-*score variation: + 0.4 ± 0.8; [t(31) = −2.874, p_two-tailed_ < 0.014]; non-dominant = raw score variation: 1 ± 1.45; *z-*score variation: + 0.38 ± 0.41 [t(18) = −4.058, *p*_corrected_ < 0.002] (Fig. 2). Patients free from seizures with impaired awareness (ILAE 1–2) showed significantly better postoperative naming performance [mean *z-*score: −0.39 95% CI (−0.91, 0.13)] compared to those with ongoing seizures [ILAE 1-2: mean *z-*score (95% CI): −0.39 (−0.91, 0.13); ILAE 3-6 mean *z-*score (95% CI): −1.52 (−2.12, −0.92); *t(*49) = 2.87, *P* < 0.006]. We hence analysed change in naming performance, from preoperative to 12 months postoperative, specifically in patients with ILAE class 1-2 seizure outcome. In this cohort of patients, naming performance significantly improved 12 months after surgery [raw score variation: 1.7 ± 3.1; *z-*score variation: + 0.48 ± 0.79; *t*(28) = −3.264, *p*_corrected_ <0.003].

A multivariable linear regression model was fitted to assess changes in postoperative from preoperative naming *z-*scores and the association with the covariates: age, sex, years of education, resection volume, epilepsy duration and use of topiramate or zonisamide. These covariates accounted for 21% of the variance in naming performance changes [*R*² = 0.210, Adjusted *R*² = 0.103] but, overall, these variables were not significantly associated with the change in naming performance [*F*(6, 44) = 1.954, *P* = 0.093]. In this model, sex and years of educations were significant predictors, with female patients showing better preservation of naming abilities after surgery [*B* = 0.431, SE = 0.191, *P* = 0.029, 95% CI (0.046, 0.816)], and higher education being associated with greater decline in naming performance [*B* = −0.110, SE = 0.054, *P* = 0.047, 95% CI (−0.218, −0.001)]. There were no statistically significant associations between other covariates and naming performance.

### Voxel-lesion symptom mapping

We performed voxel-lesion symptom mapping to evaluate if there were specific cortical areas involved in picture naming outcome. There was no significant association between resected cortical regions and language performance.

### Disconnectome analyses

Non-significant results can be found in the Supplementary material.

#### Tractwise voxel-based disconnectome analysis

**Figure 3:**
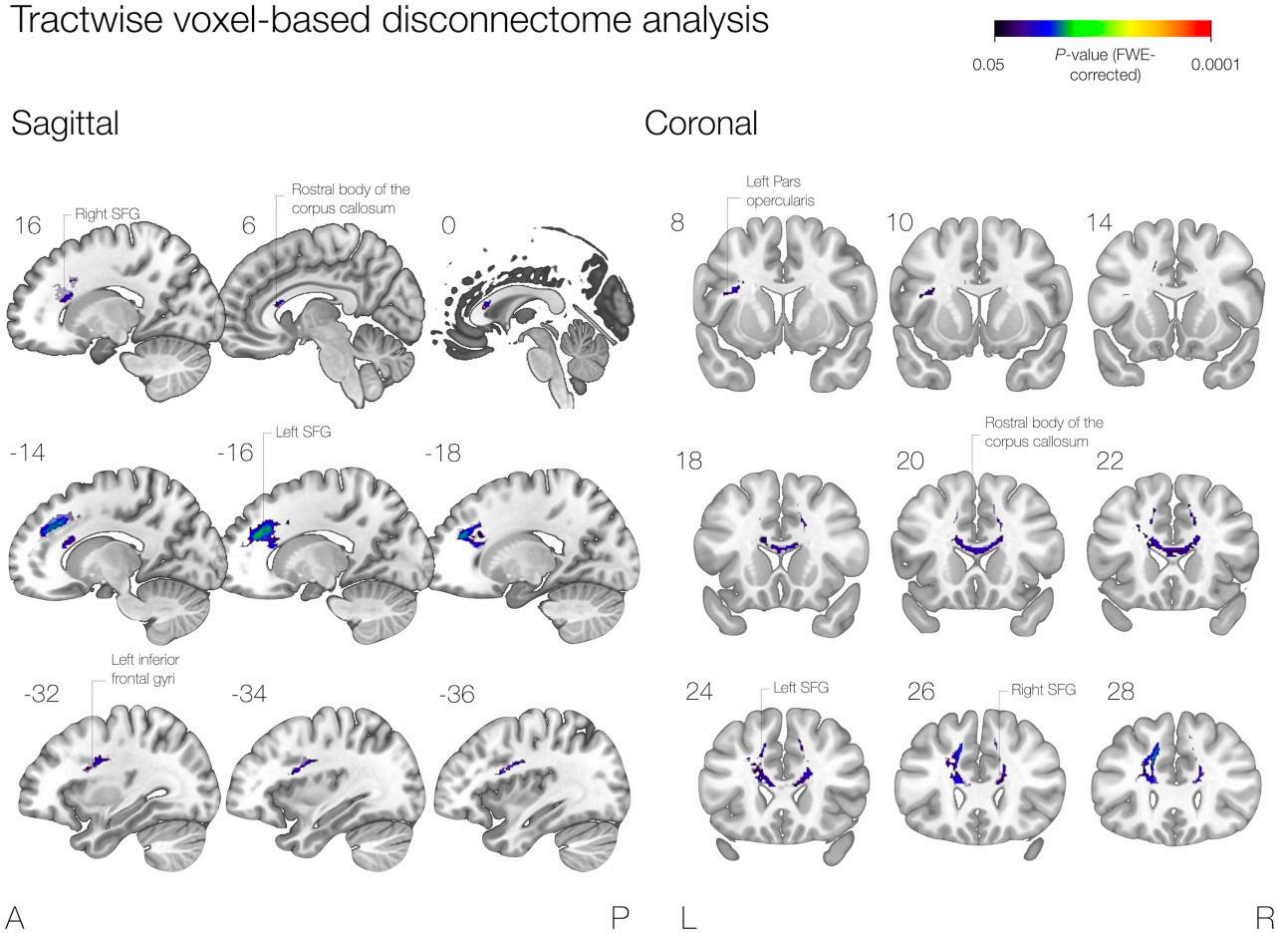
**Tractwise voxel-based disconnectome analysis.** A permutation test of resected voxels significantly associated with language improvement is shown in the coronal and sagittal planes. Significant voxels include the rostral body of the corpus callosum and projections to the inferior frontal and superior frontal gyri and represent locations that, disconnected, were associated with improved naming in this cohort.

No voxel or tract lesioning was associated with language decline at 12 months. Conversely, the permutation tests highlighted voxels associated with language improvement at the level of the rostral body of the corpus callosum, the superior frontal gyri and the left inferior frontal gyrus (Fig. 3).

#### Tractography analysis

**Figure 4:**
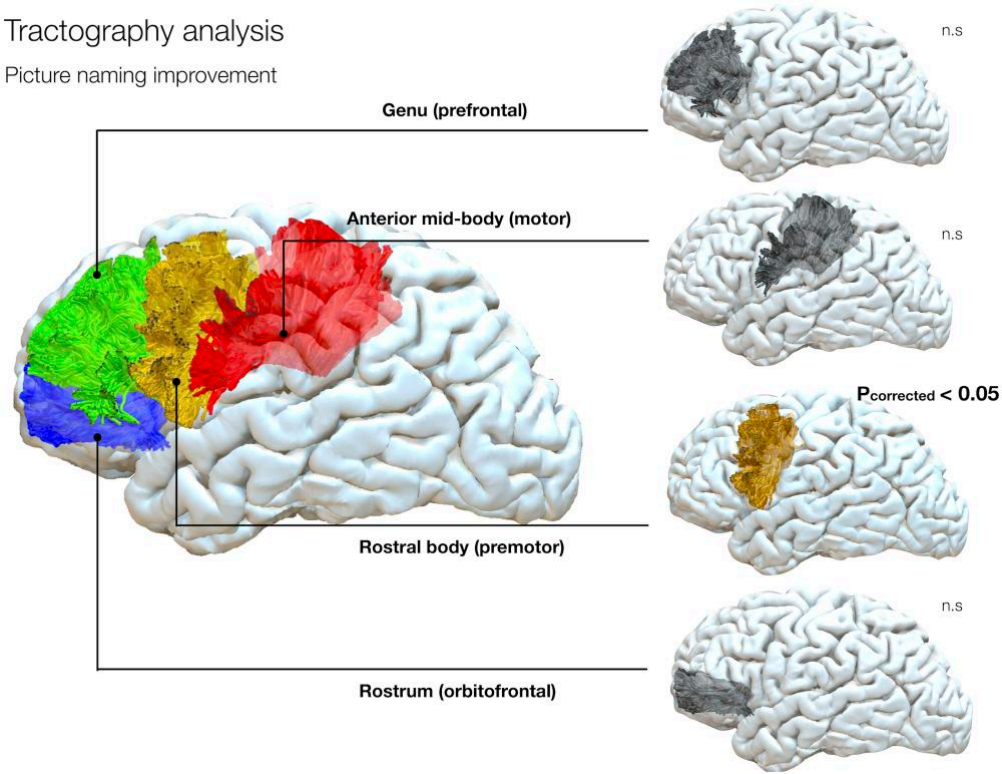
**Tractography analysis.** The corpus callosum in its frontal projections is shown. A linear regression model showed that disconnection of a larger proportion of fibres of the rostral body is associated with language improvement. Statistical tests based on model parameters are reported with corrected *P*-values. *Blue: Rostrum; Green: Genu; Yellow: Rostral body; Red: Anterior midbody.*

Following the results obtained from the disconnectome analysis, we tested the involvement of the corpus callosum in a cohort of 21 patients with preoperative tractography. Percentage of callosal disconnection for each subject can be found in Table 3. Language improvement was associated with a greater disconnection of fibres passing through the rostral body of the corpus callosum [*R*^2^ = 0.29; *F*(1,19) = 7.760; ß=0.536; *p*_corrected_ < 0.05; 95%CI = −0.22, −0.003] connecting superior frontal, posterior middle frontal gyri and inferior frontal gyri bilaterally (Fig. 4).

**Table 3:** Percentage of callosal disconnection according to callosal segments

Patient	Anterior corpus callosum	Rostrum (orbitofrontal)	Genu (prefrontal)	Rostral body (premotor)	Anterior midbody (motor)
1	69.62	100.00	100.00	75.43	0.00
2	3.78	0.00	0.05	14.95	0.00
3	0.00	0.00	0.00	0.00	0.00
4	0.98	0.00	0.11	3.31	0.35
5	13.14	0.00	0.61	92.14	0.01
6	2.65	0.00	0.00	12.47	2.25
7	0.00	0.00	0.00	0.00	0.00
8	18.16	0.00	0.75	73.12	0.00
9	10.02	0.00	3.34	50.24	1.04
10	16.58	0.00	1.44	75.79	0.06
11	4.00	0.00	6.29	4.37	0.00
12	59.87	46.52	100.00	100.00	0.00
13	30.29	0.00	42.44	63.52	0.81
14	80.76	70.13	99.98	59.10	0.00
15	2.35	0.00	0.00	4.94	20.09
16	5.93	0.00	3.12	36.20	0.27
17	3.86	0.00	1.25	9.77	6.29
18	59.33	0.00	89.22	64.13	0.00
19	37.77	51.79	98.70	0.01	0.00
20	16.48	0.00	86.79	0.00	0.00
21	0.00	0.00	0.00	0.00	0.00

## Discussion

Frontal lobe epilepsy surgery is performed with the aim of seizure freedom. Our results, however, indicate that frontal lobe resections may also improve language: our patients had significantly improved naming performance after surgery. This occurred in both hemispheres, with a stronger, albeit not-significant, effect in the non-dominant hemisphere. Critically, postoperative performance was associated with seizure reduction, suggesting that ongoing epileptic discharges impact normal function and stopping these may underlie its restoration. As language improvement was linked to disconnection of the corpus callosum, a known target to obtain seizure reduction,^9^ transection of commissural fibres may allow for language improvement by decreasing epileptic travelling waves between hemispheres. This novel framework suggests a need to refine the design of frontal lobe epilepsy surgery as both epilepsy and cognitive outcomes may improve after resection.

### Frontal lobe resection and improved picture naming

The ability to name is fundamental to verbal communication. This is particularly relevant for frontal lobe surgery, as this is considered the central hub for speech production.^10^ In our series, naming significantly improved after surgery: no patient showed a naming decline after 1 year and five patients (10%) showed a significant improvement as judged by RCIs. Although not specifically associated with seizure outcome, preliminary evidence for naming improvement was provided by Busch and colleagues. They showed naming improvement in up to 14% of patients after frontal lobe surgery, although 7% patients in the cohort declined leading to an effect size of −0.28.^7^

Data indicating language improvement after surgery may, at first glance, oppose the long-standing role of the frontal lobe in speech articulation and require careful interpretation. First, it is important to point out that the dominant dorsal and ventral laryngeal areas in the precentral gyrus were never resected in our cohort. While Broca’s area in the pars opercularis is no longer considered critical for speech articulation,^39,40^ evidence from awake surgery has highlighted that the ventral precentral cortex, overlapping dorsal and ventral laryngeal area, underlies speech production^39,41^ and damage to this may cause non-fluent aphasia.^42^ As such, preservation of this cortical hub may have prevented non-fluent aphasia, allowing for picture naming to remain intact, or even improve.

Naming, however, necessitates access to stored lexical knowledge, in addition to the sensorimotor system required to articulate. An area for multimodal semantic knowledge, comprising lexicon, has been located in the ventral temporal lobe (middle inferior temporal and fusiform gyri).^43^ Damage to this temporal area or its connections causes permanent anomia irrespective of pathology, as has been shown in patients suffering from epilepsy, tumours or neurodegeneration.^14,16,44^ Critically, analysis of neuropsychological performance in individuals with epilepsy indicates a dissociation between deficits in temporal versus frontal lobe epilepsy: naming disturbances characterize temporal lobe epilepsy—in keeping with a degradation of semantic knowledge^43^—while frontal lobe epilepsy is associated with deficits in executive function.^29^ Our results add inference to this dissociation, suggesting that naming may be preserved—and potentially improved—after frontal lobe surgery, as soon as sufficient seizure reduction is achieved.

To appropriately capture variation in naming performance, we used both reliable change indices (RCIs) and changes in *z-*scores. RCIs allow to evaluate performance in the individual patient without using normative data that in pathological conditions such as epilepsy, may hinder naming evaluation because of normalization to normative healthy data. Furthermore, when directly comparing RCIs with a confidence interval of 80% and a *z-*score variation of 1 standard deviation, it has been shown that *z-*scores may overestimate improvement and underestimate or overestimate decline,^44^ and therefore the use of 1 standard deviation as an evaluation of performance change should be cautioned.^44^ On the other hand, RCIs rely on consistent test-retest performance on an individual, but there may be benefits in comparing this to an overall performance in a cohort. It can be argued that a change of 4 items on a generally averaged performance is not the same that when the performance is very impaired or very advanced, meaning that RCIs may be over-conservative on some instances and can benefit in comparison with a normative cohort. In view of this issue, we used both measures, which showed results in a similar direction.

### Seizure reduction and callosal disconnection

Current theories propose that seizure-generating networks may disrupt appropriate behaviour through ongoing aberrant electrical activity^4^ or cause normal brain to alter its firing patterns to decrease epileptic activity at the expense of cognition.^38^ It has been shown that non-lesional epileptic tissue is capable of physiological responses and these are impaired during epileptic discharges causing the region to be in a ‘cognitive refractory state’.^4^ Similarly, evidence suggests that healthy brain may actively suppress discharges from the epileptogenic zone,^45^ potentially by altering its firing pattern at the expense of cognition.^38^ In line with this, our data suggest that seizure reduction is associated with picture naming performance, and that performance is significantly better for those cases in which no seizures with impaired awareness occur. Hence, epilepsy may interfere with, or ‘exert a brake’ upon normal cognitive functioning, in which case disconnecting the epileptogenic network may stop the interference or ‘release the brake’ and restore normal function. This proposal is supported by results of a large paediatric cohort who underwent epilepsy surgery and in which it has been shown that resection may not only halt further cognitive decline but also be at the basis of functional recovery to expected neuropsychological trajectories, meaning that brain networks may recover function.^46^ Our data suggest that this phenomenon may not be restricted to children, and adults may also benefit from seizure reduction to recover cognition.

Our results indicate that disconnection of the corpus callosum may be associated with picture naming improvement. Since the 1940s, callosal disconnection has been advocated for seizure reduction, performed as a standalone procedure in callosotomy or as a component of wider disconnection in functional hemispherotomy. Accordingly, and in line with our results showing that language performance scales linearly with seizure reduction, we speculate that callosal disconnection may contribute to language improvement through seizure reduction by limiting seizure spread via commissural fibres to the unaffected hemisphere.^3,6^ This may be particularly relevant in those individuals in whom seizures do not originate in language areas, but language impairment is caused by propagation of epileptic activity. Similarly, it is possible that for some frontal epileptic networks that involve both hemispheres, with oscillation and amplification involving callosal fibres,^47,48^ the transection of which results in reduced interference with function of language networks. As a result, function may improve after disconnection, as previously theorized in split-brain patients.^49^ On this aspect, it is noteworthy that language improvement occurred in both hemispheres, suggesting a role for seizure spread in both dominant and non-dominant resections.

To conclude, stopping epileptic travelling waves to language regions may directly induce language improvement. A second form of improvement, however, may occur over time since seizures not only affect normal brain activity but may also impair cerebral reorganization and plasticity.^46,50^While these mechanisms for recovery have just been recently elucidated in patients reoperated for low grade glioma^51^ and it is unclear how this would translate in epilepsy surgery,^52^ it may be particularly relevant for surgery in the frontal lobe as frontal language regions have shown high plastic potential at a cortical level.^51,53^

## Limitations

This study has limitations. Our cohort of 51 patients is relatively small although it is the largest published cohort of operated patients for frontal lobe epilepsy with neuroimaging. Moreover, our cohort is composed of patients with heterogeneous causes of epilepsy, which may have different degrees of neuroplasticity and therefore neuropsychological outcome. As an example, brains with low grade gliomas have high plastic potential,^53^ but it is unclear whether other epileptogenic lesions induce similar patterns of network reorganization impacting on language function. Further, different scanners have been used since the data acquisition has lasted three decades, which may have impacted imaging evaluation. This is particularly relevant for diffusion imaging as a smaller voxel size may imply a higher number of streamlines evaluated. While we used percentage of streamlines disconnected to correct for this, we cannot exclude that the overall number of streamlines may produce some degree of inconsistency among images evaluated. Most importantly, in this study we have evaluated outcome for picture naming: while neuropsychological assessment in clinical settings is necessarily constrained to simple, broad batteries of tests, picture naming does not cover the whole domain of language function. As other facets that are critical to language have not been evaluated, the role that callosal disconnection may have on these remains to be clarified. Beside this, language is only one of the aspects that involve frontal lobe function, and others such as executive planning, motor function, attention and working memory are not tested in our dataset. Considering this, while picture naming may improve, a more fine-grained neuropsychological examination is critical to comprehensively understand the role of frontal lobe surgery in functional recovery.

## Conclusions

The impact of frontal lobe epilepsy surgery on language is poorly understood, as surgery can damage eloquent structures, but interruption of epileptic discharges may support improvement. In this study, we showed that language improvement is associated with seizure reduction, and this may occur irrespective of language dominance or operated hemisphere. As anterior callosal disconnection, a target for seizure reduction, was associated with language improvement, our results suggest that freeing the language network from epileptic activity may support recovery. This novel framework is important for designing frontal lobe epilepsy surgery in both dominant and non-dominant hemispheres, as targeted resection and disconnection with adequate subcortical preservation may enable recovery of language function, particularly in those patients with preoperative deficits.

## Supplementary Material

fcaf317_Supplementary_Data

## Data Availability

Anonymized data that support the findings of this study are available from the first author or corresponding authors upon reasonable request.
