# Supplementary material for: Impact of anterior callosal disconnection on picture naming in frontal lobe epilepsy surgery
Source: Brain Commun. 2025 Sep 3;7(5):fcaf317. doi: 10.1093/braincomms/fcaf317 (PMC12417999; doi:10.1093/braincomms/fcaf317)
Supplement: fcaf317_Supplementary_Data [file fcaf317_supplementary_data.pdf]

Supplementary Material

Voxel-based and tractography analysis pipeline

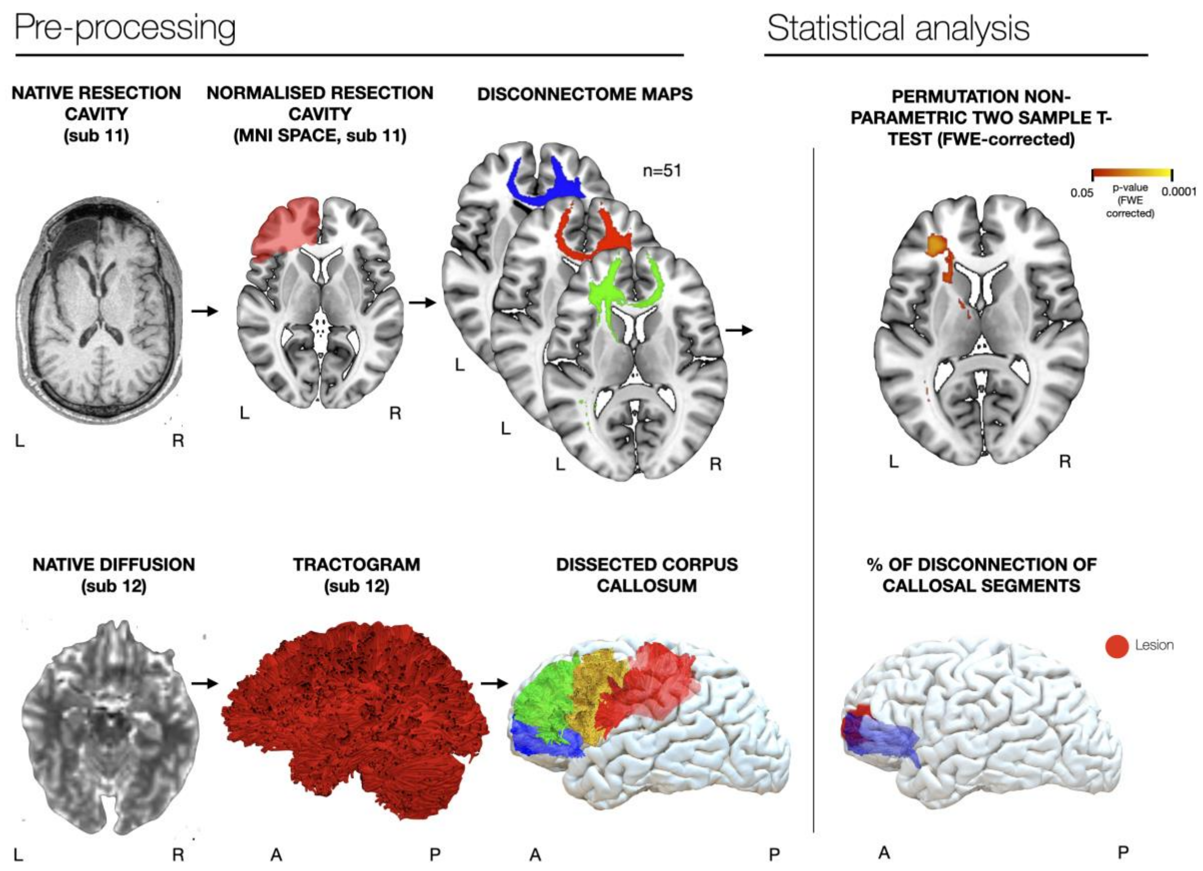

Supplementary Figure 1. **Processing pipelines.** Above the tractwise voxel-based pipeline is shown. Below, the tractography pipeline is shown.

The voxel-lesion symptom mapping was performed as follows: first, a mask of the resection cavity was performed using ITK-snap (http://www.itksnap.org/pmwiki/pmwiki.php). Then, each resection mask was normalised to the MNI space (1 mm) using an enantiomorphic coregistration as implemented in SPM12 (https://www.fil.ion.ucl.ac.uk/spm/software/spm12/). Once this was performed, the normalised cavities were tested using FSL randomise using a non-parametric permutational two sample t-test using a differential of z-scores (postoperative z-score minus preoperative z-score).

Tractography processing and reconstruction

Anatomical priors

Segments of the corpus callosum were reconstructed with an updated version of anatomically targeted – automated tractography[1] which included anatomical priors from the human connectome project 7T dataset.[2] Briefly, the geodesic information flow (GIF)[3] parcellation was used to extract cortical regions which were used as seed / termination points for tractography. Anatomically constrained tractography (ACT)[4] using hybrid surface and volume segmentation in MRtrix3[5] was performed using second-order integration over fibre orientation distribution probabilistic fibre tracking algorithm[6] selecting a maximum of 5000 streamlines from 30 million seeds. Tractography was performed twice, switching the seed and termination cortical region-of-interest (ROI). Fibre bundles were then converted to probabilistic maps, thresholded at a value of 0.01, and used as an exclusion criterion to remove spurious streamlines. Fibre bundles were inspected to ensure accurate reconstruction and manual exclusion masks were used to remove spurious streamlines. The cleaned tractograms were then transformed to MNI space, converted to a binary mask, and dilated by 1mm to account for coregistration errors.

Supplementary Table 1 – Cortical terminations for segments of the corpus callosum

Fibre Bundle	Seed ROI	Termination ROI
**CC1 (Rostrum)**	Left medial frontal cortex,<br>Left frontal pole,<br>Left medial orbital gyrus,<br>Left gyrus rectus	Right medial frontal cortex,<br>Right frontal pole,<br>Right medial orbital gyrus,<br>Right gyrus rectus
**CC2 (Genu)**	Left anterior-half middle frontal gyrus,<br>Left anterior-half superior frontal gyrus,<br>Left lateral orbital gyrus,<br>Left pars triangularis,<br>Left pars orbitalis,<br>Left frontal pole	Right anterior-half middle frontal gyrus,<br>Right anterior-half superior frontal gyrus,<br>Right lateral orbital gyrus,<br>Right pars triangularis,<br>Right pars orbitalis,<br>Right frontal pole
**CC3 (Rostral body)**	Left posterior-half middle frontal gyrus,<br>Left posterior-half superior frontal gyrus,<br>Left frontal operculum,<br>Left pars opercularis,<br>Left supplementary motor cortex	Right posterior-half middle frontal gyrus,<br>Right posterior-half superior frontal gyrus,<br>Right frontal operculum,<br>Right pars opercularis,<br>Right supplementary motor cortex
**CC4 (Anterior midbody)**	Left central operculum,<br>Left precentral gyrus,<br>Left medial precentral gyrus	Right central operculum,<br>Right precentral gyrus,<br>Right medial precentral gyrus

Tractography in the epilepsy patient cohort

To reconstruct the fibre bundles of interest, the MNI HCP 7T masks were transformed to native space of each patient using EasyReg.[7] The same cortical regions and tractography parameters that were used to create the anatomical priors were used to reconstruct the fibre bundles; the 7T anatomical priors were used as an inclusive mask. Any streamlines exiting the mask were discarded. Each fibre bundle was manually inspected to ensure accuracy.

Tractography results

Rostrum

There was no significant association between rostral disconnection and picture naming outcome (R^2=0.55; $F(1,19)$=0.999; ß=0.236; $p_{two\text{-}tailed} < 0.332$; 95%CI = -0.22:0.008)

Genu

There was no significant association between genual disconnection and picture naming outcome (R^2=0.58; $F(1,19)$=1.163; ß=0.240; $p_{two\text{-}tailed} < 0.294$; 95%CI = -0.14:-0.004)

Anterior mid-body

There was no significant association between anterior mid-body disconnection and picture naming outcome (R^2=0.31; $F(1,19)$=0.579; ß=0.536; $p_{two\text{-}tailed} < 0.456$; 95%CI = -0.59:0.125)

References

1. Binding LP, Dasgupta D, Taylor PN, et al. Contribution of White Matter Fiber Bundle Damage to Language Change After Surgery for Temporal Lobe Epilepsy. *Neurology*, 2023:10.1212/WNL.0000000000206862. doi:10.1212/WNL.0000000000206862
2. Vu AT, Auerbach E, Lenglet C, et al. High resolution whole brain diffusion imaging at 7 T for the Human Connectome Project. *Neuroimage*. 2015;122:318-331.
3. Cardoso MJ, Modat M, Wolz R, et al. Geodesic information flows: spatially-variant graphs and their application to segmentation and fusion. *IEEE Trans Med Imaging*. 2015;34(9):1976-1988.
4. Smith RE, Tournier JD, Calamante F, Connelly A. Anatomically-constrained tractography: improved diffusion MRI streamlines tractography through effective use of anatomical information. *Neuroimage*. 2012;62(3):1924-1938.
5. Tournier JD, Smith R, Raffelt D, et al. MRtrix3: A fast, flexible and open software framework for medical image processing and visualisation. *Neuroimage*. 2019;202:116137.
6. Tournier JD, Calamante F, Connelly A. Improved probabilistic streamlines tractography by 2nd order integration over fibre orientation distributions. In: *Proceedings of the International Society for Magnetic Resonance in Medicine*. Vol 1670. John Wiley & Sons, Inc, New Jersey; 2010.
7. Iglesias JE. A ready-to-use machine learning tool for symmetric multi-modality registration of brain MRI. *Sci Rep*. 2023;13(1):6657.
